# Digitization inequality: how robotization shapes gendered perceived pay fairness in China

**DOI:** 10.3389/fpsyg.2025.1627690

**Published:** 2025-08-22

**Authors:** Li Zhang, Xianqing Tu, Xin Yong

**Affiliations:** ^1^School of Economics, Guangdong University of Finance and Economics, Guangzhou, China; ^2^School of Public Administration, Xiangtan University, Xiangtan, China

**Keywords:** robotization, perceived pay fairness, gender, manufacturing industry, China

## Abstract

**Objective:**

This study aims to examine the gendered effects of robotization on workers’ perceived pay fairness (PPFs) in the Chinese manufacturing industry. It specifically investigates how robotization is associated with gender disparities in PPFs and explores the mediating roles of wage dynamics and skill development in shaping these outcomes.

**Method:**

We analyzed survey data from 28,470 manufacturing workers in Guangdong, China, using ordinary least squares regression to examine the association between robotization and perceived pay fairness. Instrumental variable techniques were used to address potential endogeneity. Mediation analyses assessed the roles of wages and skill levels.

**Results:**

Robotization is positively associated with perceived pay fairness among workers, with a stronger effect for men than for women. This gender gap is mainly explained by greater wage gains for men. Wage growth significantly mediates the relationship between robotization and PPFs for both genders, but the effect is weaker for women. While robotization raises skill levels for both men and women, only among men do increased skills modestly reduce the positive effect of robotization on PPFs; for women, skill development shows no significant impact.

**Discussion:**

These results highlight persistent gender disparities in the benefits of robotization, with male workers reporting greater perceived gains in pay fairness. The weaker effect for female workers is mainly due to smaller wage increases and limited impact of skill improvements. The study suggests that policies should address gender wage gaps in robotized workplaces and support women in recognizing and utilizing their skills.

## 1 Introduction

The rise of industrial robots has profoundly reshaped labor markets, highlighting significant gender disparities in employment outcomes (Aksoy et al., 2020; Ge and Zhou, 2020; Tejani and Kucera, 2021). Although existing research primarily focuses on objective labor market metrics such as employment rates (Tejani and Kucera, 2021) and wage inequality (Aksoy et al., 2020; Ge and Zhou, 2020), limited attention has been paid to subjective aspects like perceived pay fairness (PPFs). Examining the implications of automation on PPF is crucial, as there is often a mismatch between objective pay fairness and employees’ perceptions of pay fairness (Paul, 2006; Pfeifer and Stephan, 2018), and these perceptions strongly shape employee behaviors, attitudes, and organizational outcomes (Abdin et al., 2020; Hosmer and Kiewitz, 2015).

This study examines how robotization influences workers’ PPF and explores gender differences in these impacts. According to Equity Theory (Adams, 1965), workers evaluate pay fairness by comparing their inputs and outputs (Okpara, 2006; Al-Zawahreh and Al-Madi, 2012). The technological changes brought about by robotization, including shifts in skill requirements, task complexity, and compensation structures, inevitably affect these internal fairness evaluations. Critically, as prior research has shown (Aksoy et al., 2020; Ge and Zhou, 2020), the impact of robotization is not gender-neutral: rather than simply raising overall productivity, robotization tends to reshape PPF in ways that reinforce or even amplify gender disparities within the workplace.

Equity Theory further suggests that perceptions of unfairness arise when employees believe their compensation does not adequately reflect their contributions, such as skills, effort, and experience (Kim et al., 2010; Collins et al., 2023). Empirical evidence demonstrates that subjective pay fairness perceptions can differ considerably from objective fairness due to variations in internal standards and comparative frameworks (Paul, 2006; Pfeifer and Stephan, 2018). For instance, studies by Pfeifer and Stephan (2018), and Jackson et al. (1992) show that women generally hold lower pay expectations and are more accepting of lower compensation relative to their contributions, possibly due to historical wage discrimination, socialization processes, or comparisons predominantly with similarly situated women in lower-paid roles. Therefore, it is essential to investigate how robotization shapes perceived pay fairness from a gender perspective.

Moreover, understanding the implications of PPFs in the context of robotization is particularly significant from an organizational behavior perspective. Numerous studies demonstrate that perceived fairness profoundly influences employee attitudes, including organizational commitment, job satisfaction, and turnover intentions (Abdin et al., 2020; Buttner and Lowe, 2016; Day, 2012; Mohrenweiser and Pfeifer, 2023). Negative perceptions of pay fairness can trigger behaviors ranging from reduced effort to absenteeism or resignation (Al-Zawahreh and Al-Madi, 2012; Eren and Demir, 2023; Perry, 1993; Sun and Deng, 2024). Therefore, analyzing how technological changes like robotization reshape PPFs is vital for organizational effectiveness and employee well-being.

In addition to directly analyzing the relationship between robotization and PPFs, this study examines the mediating roles of wages and skill levels. Within Equity Theory’s framework, wages constitute a critical work output (Tekleab et al., 2005; Collins et al., 2023), whereas skill levels represent an essential work input (Lee et al., 1999). By exploring how these mediating factors differ by gender, the analysis aims to reveal distinct pathways through which robotization influences wage structures, skill requirements, and ultimately, perceptions of pay fairness.

To empirically investigate these issues, this study utilizes data from a large survey of manufacturing workers in Guangdong province, China—a prominent region in global manufacturing and rapid robotization. China’s aggressive push toward industrial robotization since the 2010s, driven by policies shifting from labor-intensive to technology-intensive manufacturing (Huang and Sharif, 2017), offers a critical context for examining gendered automation impacts. Our analysis proceeds in several steps. First, we use ordinary least squares (OLS) regression to examine how robotization influences workers’ PPFs. Second, we incorporate an interaction term between robotization and gender to assess potential gender differences in this relationship. Third, we conduct instrumental variable (IV) analysis and a series of robustness checks to address endogeneity and ensure the stability of our results. Fourth, we explore the mediating roles of hourly wages and skill levels in the relationship between robotization and PPFs for male and female workers. By highlighting robotization as a critical factor, this study challenges the notion of technological neutrality and reveals how automation can reinforce and reproduce gender inequalities in the workplace.

## 2 Theoretical background and hypotheses

### 2.1 Robotization, PPFs, and gender differences

Equity theory provides a crucial theoretical foundation for analyzing the formation of workers’ PPFs. According to this theory, workers develop their PPFs by evaluating the balance between the inputs they contribute to their jobs—such as working hours, skills, experience, effort, and social connections—and the corresponding rewards, primarily wages (Adams, 1965). When employees perceive that their compensation fairly reflects their contributions, their PPFs improve. Conversely, when they believe their pay is disproportionately low compared to their inputs, feelings of inequity and dissatisfaction arise.

Building on this perspective, robotization has the potential to reshape workers’ PPFs by changing both job inputs and outputs. Empirical research in the Chinese manufacturing industry suggests that robotization can reduce job inputs by improving working conditions, lowering workloads, and mitigating workplace hazards, while simultaneously increasing job output through wage growth (Hou et al., 2020; Sun and Deng, 2020) and job satisfaction (Du et al., 2024). For example, industrial robots have been shown to enhance work environments by reducing workers’ exposure to hazardous conditions such as extreme temperatures, dust, and noise (Hou et al., 2020; Liu et al., 2024). Robots also take over physically demanding, repetitive, and dangerous tasks, including heavy lifting, stacking, and working at heights, alleviating physical strain and reducing the risk of injury (Sun and Deng, 2020). Additionally, research suggests that robotization has generally led to wage increases (Hou et al., 2020), and these improvements in working conditions have contributed to higher job satisfaction (Du et al., 2024). Within the equity theory framework, such changes can be interpreted as either lowering the “cost” side (inputs) for workers or increasing the “reward” side (outputs). Both mechanisms are likely to foster higher PPF: workers feel that, with less hardship and/or higher pay, their work relationship is more equitable, thus improving their overall work attitudes and satisfaction.

While equity theory emphasizes that perceived fairness stems from the comparison between individual inputs and received outputs (Adams, 1965), it also highlights the importance of subjective reference points, which are shaped by social norms and prior experiences (Jackson and Grabski, 1988; Pfeifer and Stephan, 2018; Khoreva, 2011). A large body of research indicates that women, especially in female-dominated sectors, tend to internalize lower pay expectations and compare themselves to lower-wage reference groups, which makes them more likely to perceive even modest pay as fair (Jackson and Grabski, 1988; Paul, 2006). Men, on the other hand, generally have higher pay expectations and benchmark their fairness standards against higher wages and more advantageous working conditions.

As a result, men are more likely to perceive pay as unfair and are also more sensitive to improvements in pay or working conditions. When robotization brings about better wages and working conditions, men are more inclined to register an increase in PPF, since these improvements help close the gap between their higher expectations and their actual rewards. For women, whose fairness perceptions are already less sensitive to under-reward and more “anchored” at a lower level due to internalized norms and lower expectations, the same improvements may not translate into equally strong increases in PPF.

Based on the above theoretical analysis, we propose the following two hypothesizes:

*Hypothesis 1*. Robotization has a positive impact on workers’ PPFs.

*Hypothesis 2*: The positive impact of robotization on PPFs is more pronounced for male workers than for female workers.

### 2.2 Mediating role of wages in the robotization-PPF relationship

In addition to its direct effect on PPFs, robotization also impacts workers’ wages, which may further contribute to gender differences in PPFs. Some studies have shown that robotization generally leads to wage growth for workers, but the extent of these gains may differ by gender (Aksoy et al., 2020; Pavlenkova et al., 2023). For example, Aksoy et al. (2020) found that while robotization increased wages for both men and women across 20 European countries, it also widened the gender wage gap. This was largely because men were more likely to hold middle-skill jobs that benefited from robotization, whereas automation reduced women’s presence in these positions. Similarly, Pavlenkova et al. (2023) found that automation, including the adoption of industrial robots, had a significantly stronger impact on male wage growth compared to female wage growth, further exacerbating the gender wage gap.

Wages, as a central outcome of work (Collins et al., 2023; Tekleab et al., 2005), play a crucial impact on workers’ PPFs. Generally, higher wages are correlated with enhanced PPFs. Therefore, wages may act as a key mediating factor in the relationship between robotization and PPFs.

However, it is important to note that the mediating role of wages in this relationship may differ across genders. Previous studies indicate that women are generally less dissatisfied or more accepting of lower pay compared to men (Callahan-Levy and Messé, 1979; Kuhn, 1987; Paul, 2006; Pfeifer and Stephan, 2018). For example, in their experimental study, Callahan-Levy and Messé (1979) reveal that for the same job, women are prone to pay themselves lower wages than men are and perceive these lower wages as fair. Similarly, Paul (2006) finds that women are less likely than men to perceive that they are unfairly paid. Pfeifer and Stephan (2018) also demonstrated that even with identical hourly wages and job characteristics, women tend to perceive their wages as fair more frequently than men do.

Therefore, while robotization may result in smaller wage increases for women compared to men, women may still be more satisfied with these smaller wage increases. As a result, the positive mediating effect of wages on PPFs in the robotization-PPF relationship may not be weaker for women compared to men, despite their lower wage growth.

Based on the above theoretical analysis, we propose the following hypothesis:

*Hypothesis 3*: Robotization increases hourly wages for both men and women, which are linked to higher PPFs for both genders.

*Hypothesis 4*: Although robotization is associated with smaller wage increases for women compared to men, this difference in wage growth does not weaken the positive effect of wages on women's PPFs relative to men.

### 2.3 Mediating role of skill levels in the robotization-PPF relationship

Several studies have demonstrated that the adoption of industrial robots increases the demand for higher-skilled workers, as robots typically take over repetitive, low-skill tasks, such as palletizing and packaging, while creating new high-skill roles in areas like software development and technical maintenance (e.g., Fierro et al., 2022). Moreover, high-skilled workers are generally better equipped to adapt to robotized work environments, as they are more capable of learning and utilizing robots effectively to enhance productivity (Dixon et al., 2021). As a result, robotization drives firms to increase their demand for high-skilled workers (Acemoglu and Restrepo, 2020; Graetz and Michaels, 2018). Empirical evidence from China supports this trend, showing that robotization encourages firms to hire more skilled workers (Tang et al., 2021).

As a key component of job input, workers’ skill levels play a crucial role in shaping their PPFs (Lee et al., 1999). Typically, higher skill levels are linked to elevated wage expectations, as workers anticipate that their enhanced abilities will be rewarded. However, when wage expectations rise without a corresponding increase in compensation, workers may perceive their pay as less fair compared to those with lower expectations. This disparity may be linked to reduced PPFs. Therefore, while robotization increases workers’ skill levels, it may also raise their wage expectations, which could potentially weaken the overall positive impact of robotization on PPFs if the expected wage increases do not materialize.

However, the mediating role of skill levels in the relationship between robotization and PPFs may differ between male and female workers. Research suggests that, due to long-standing gender discrimination in the labor market, women often internalize gender biases, undervalue their own skills, and set lower wage expectations compared to men (Jackson et al., 1992; Pelham and Hetts, 2001). In contrast, men tend to overestimate their abilities due to overconfidence, resulting in higher wage expectations and lower PPFs (Pelham and Hetts, 2001). For instance, Jackson et al. (1992) found that men generally perceive themselves as having higher business acumen, resulting in greater wage expectations than women, which can cause lower PPFs at the same wage level.

Furthermore, technologies such as industrial robots, often associated with masculine work culture (Wajcman, 1991), may intensify these differences. Women, more likely to undervalue their skills in a robotized environment, may continue to set lower wage expectations, even as robotization enhances their skill levels (Beyer, 1990; Correll, 2001). As a result, increases in skill levels may have a limited role in reducing the positive impact of robotization on PPFs among female workers, as their wage expectations remain modest. On the other hand, men, who typically exhibit greater confidence in their skills, are likely to set higher wage expectations as their skill levels increase through robotization. However, this rise in wage expectations may diminish the positive impact of robotization on their PPFs. In other words, while robotization enhances both men’s skills and wages, the accompanying increase in wage expectations may reduce the perceived fairness of their pay as these expectations rise. Therefore, while robotization enhances the skill levels of both men and women, the negative impact of increasing skill levels on PPFs is likely to be more pronounced for men than for women due to differing wage expectations.

Based on the above theoretical analysis, we propose the following hypothesis:

*Hypothesis 5*: Robotization increases the skill levels of male workers, which in turn reduces the positive impact of robotization on their PPFs.

*Hypothesis 6*: Robotization also increases the skill levels of female workers, but the mediating effect of skill levels in weakening the positive impact of robotization on PPFs is less pronounced for female workers compared to male workers.

## 3 Data, variables and models

### 3.1 Data

The data used in this study are drawn from a large-scale survey of employee working conditions conducted in Guangdong province in 2022. Guangdong is the manufacturing hub in China. In 2022, the total output value of Guangdong’s manufacturing industry exceeded RMB16 trillion (approximately USD2.38 trillion), accounting for one eighth of the nation’s overall output (Guangdong Provincial Taxation Bureau, 2023). Guangdong is also at the forefront of efforts to upgrade the manufacturing industry by embracing advanced technologies, including industrial robots. In 2015, the Guangdong provincial government took an important step by allocating a substantial fund of USD150 billion to support firms’ investment in automation technologies and foster innovation in the field of robotics (Yang, 2017).

The survey was conducted by a group of scholars and students from South China Normal University in Guangdong province. For data collection, workers were invited to participate by filling out e-questionnaires via a survey link accessible on their mobile phones. To broaden participation, the research team encouraged workers to share the survey link with their colleagues in Guangdong through popular social platforms such as QQ and WeChat. From July 4 to August 5, 2022, a total of 34,103 e-questionnaires were completed. After careful data screening, 1,226 incomplete or irrelevant responses were excluded. This filtering process resulted in a dataset of 32,877 valid questionnaires, representing a response rate of 96.41%. As our study focuses on the manufacturing industry, responses from non-manufacturing sectors were excluded, yielding a final dataset of 28,470 workers in Guangdong’s manufacturing industry.

Table 1 displays an overview of the socio-demographic and job-related characteristics of the respondents. The findings reveal that 54.95% of the respondents were male and 45.05% were female. On average, the respondents were 35.96 years old, with an average employment tenure of 7.09 years. A notable majority (72.70%) held rural *hukou* status.^1^ In terms of education, the majority of the respondents had completed junior high school (30.24%) or senior high school (29.45%). The reported average hourly wage was RMB23.37 (approximately USD3.47).

**Table 1:** Sample distribution.

Individual-level variable	Mean/Perct.	Firm-level variable	Mean/Perct.
PPFs	3.15	No robotization	31.27%
Male PPFs	3.17	Limited robot adoption	51.95%
Female PPFs	3.12	Moderate robotization	13.75%
Respondents without a college degree	3.07	Extensive robotization	3.04%
Respondents with a college degree	3.17	Small firm	17.61%
Age (years)	35.96	Medium-sized firm	48.18%
Aged 15–25	15.06%	Large firm	34.21%
Aged 26–35	35.12%	State-owned firm	32.38%
Aged 36–45	32.44%	Privately owned firm	40.87%
Aged 46–55	15.59%	Firms with other ownership	26.75%
Aged 55–62	1.79%	Textile, apparel, leather, and footwear manufacturing industry	8.69%
Male	54.95%	Computer and electronics manufacturing industry	22.40%
Female	45.05%	Machinery, automotive, and equipment manufacturing industry	12.85%
Elementary school education or below	3.98%	Food and beverage manufacturing industry	6.48%
Junior high school education	30.24%	Furniture and office supply manufacturing industry	2.05%
High school or vocational school education	29.45%	Metal manufacturing industry	9.27%
Associate degree	19.33%	Chemical, rubber, and plastic manufacturing industry	6.28%
Undergraduate education	16.14%	Household appliance and electrical manufacturing industry	5.07%
Graduate education	0.87%	Packaging and printing industry	2.07%
Urban *hukou*	27.30%	Other manufacturing industries	24.84%
Rural *hukou*	72.70%		
Hourly wage (RMB)	23.37		
Length of employment (years)	7.09		
Occupational skill level	1.89		

Moreover, the distribution across firm sizes indicates that 17.61% of the respondents worked in small firms, 48.18% in medium-sized firms, and 34.21% in large firms.^2^ The data reveal that 40.87% of the respondents were employed by privately owned firms, 32.38% by state-owned firms, and 26.75% by firms with other types of ownership, including foreign, Hong Kong, Taiwanese, and Macanese ownership.

### 3.2 Variables

#### 3.2.1 Dependent variable

The dependent variable is the workers’ PPFs. Following the measurement approach used by Kim et al. (2010), PPF was assessed using a survey question that asked respondents to rate how fair they considered their pay to be on a five-point Likert scale (1 = not at all fair, 5 = very much fair). Higher values indicate a higher PPF. The average PPF among the respondents is 3.15, with men scoring higher at 3.17 than women at 3.12. An independent samples t-test confirmed that the difference in PPF between men and women was statistically significant (*t* = 3.63, df = 28,493, *p* = 0.0003).

#### 3.2.2 Independent variable

The key explanatory variable is robotization, measured by a scale developed by the authors to capture workers’ direct observations and perceptions of industrial robot adoption in their workplaces. Respondents were asked to assess the extent to which industrial robots have been adopted in their workshops. This variable is categorical: 1 indicates no adoption, 2 limited adoption, 3 moderate adoption, and 4 extensive adoption. Higher values indicate wider use of industrial robots, and this variable is treated as continuous. The descriptive analysis shows that 31.27% of the respondents’ workplaces have not adopted industrial robots, 51.95% have limited adoption, 13.75% have a moderate extent of adoption, and 3.04% have extensively adopted robots.

#### 3.2.3 Mediating variables

Hourly wages are calculated by dividing the average weekly income of surveyed workers by their average weekly working hours.

As to the variable of skill levels, we use occupational skill certificates as a metric of the surveyed workers’ occupational skill levels. In China, skill levels are assessed by firms and social organizations endorsed by the Human Resources and Social Security Department. Upon successful assessment, workers receive a certificate indicating their categorization into one of five grades: junior worker, intermediate worker, senior worker, technician, or senior technician. The occupational skill levels of the surveyed respondents are divided into six categories, with 1–6 representing hold no skill certificate to hold a senior technician certificate, respectively.

#### 3.2.4 Control variables

The study controlled for variables related to individuals’ sociodemographic characteristics and job-related characteristics. The sociodemographic characteristics are sex, age, *hukou* (household registration) status, length of employment, and education levels.

The job-related characteristics are firm ownership type, firm size, and relative hourly wage. Among them, firm ownership is categorized as state, private, or other ownership (such as foreign, joint Sino–foreign, Hong Kong, Taiwanese, and Macanese ownership). Firm size is categorized as small, medium, or large.^3^ We also control for the relative hourly wage, which is calculated by dividing the individual hourly wage by the average hourly wage of the sample.

Additionally, fixed effects for manufacturing sub-industries and cities were included to account for systematic differences across these dimensions.

### 3.3 Models

We use the following OLS regression model to test Hypothesis 1:


(1) $${\mathrm{PPF}}_{\text{ijsc}}=\alpha +\varepsilon_{1}{\text{robot}}_{i}+\varepsilon_{2}V_{i}+\varepsilon_{3}V_{j}+{Ø}_{s}+{Ø}_{c}+\varepsilon_{i}$$

In Equation 1, PPF*_ijsc_* denotes the PPF score of individual *i* in job *j*, industry *s*, and city c. The key explanatory variable, ${\text{robot}}_{i}$ , represents the level of robotization exposure for individual *i*. $V_{i}$ and $V_{j}$ are vectors of individual-level and job-level control variables, respectively. ${Ø}_{s}$ and ${Ø}_{c}$ capture industry and city fixed effects. $\varepsilon_{i}\;$ is the error term.

To examine Hypothesis 2, we further estimate the following interaction model:


(2) $$\begin{array}{l} {\mathrm{PPF}}_{\text{ijsc}}=\alpha +\varepsilon_{1}{\text{robot}}_{i}+\pi \text{gender}+{\text{μrobot}}_{i}\times {\text{gender}}_{i}+\\ \varepsilon_{2}V_{i}+\varepsilon_{3}V_{j}+{Ø}_{s}+{Ø}_{c}+\varepsilon_{i} \end{array}$$

In Equation 2, *gender_i_* is a dummy variable indicating the gender of worker *i*, and *robot_i_* × *sex_i_* is an interaction term that captures whether the effect of robotization differs between men and women. The coefficient μ represents the gender-specific effect of robotization on perceived pay fairness.

## 4 Results

### 4.1 OLS analysis of the relationship between robotization, workers’ PPFs, and gender differences

Table 2 presents the association between robotization and workers’ PPFs. In Model 1, the baseline regression shows that robotization is significantly positively associated with workers’ PPFs, with results significant at the 1% level (*p* < 0.01). Specifically, as the degree of robotization progresses from no adoption to extensive adoption, each higher level is associated with a 12.5% increase in PPFs. This relationship remains robust even after controlling for workers’ sociodemographic and job-related characteristics in Models 2 and 3, lending support to Hypothesis 1.

**Table 2:** Relationship between robotization and workers’ PPFs and associated gender differences.

	Model (1): Baseline model	Model (2): With controls	Model (3): With gender dummy	Model (4): With interaction
Variables	PPFs	PPFs	PPFs	PPFs
Robotization	0.125***	0.129***	0.130***	0.184***
	(0.00744)	(0.00750)	(0.00751)	(0.0224)
Female (male = 0)			0.0301***	0.103***
			(0.0112)	(0.0304)
Robotization × Female				−0.0377**
				(0.0147)
Education		−0.00718***	−0.00701***	−0.00697***
		(0.00223)	(0.00223)	(0.00223)
Length of employment		−0.0222***	−0.0223***	−0.0223***
		(0.00122)	(0.00122)	(0.00122)
Age		0.00179**	0.00180**	0.00185**
		(0.000753)	(0.000753)	(0.000753)
*Hukou* status		0.00686	0.00499	0.00459
		(0.0132)	(0.0132)	(0.0132)
Relative hourly wage		0.323***	0.327***	0.326***
		(0.0112)	(0.0113)	(0.0113)
Private ownership (state ownership = 0)		−0.0841***	−0.0844***	−0.0843***
		(0.0135)	(0.0135)	(0.0135)
Other ownership (state ownership = 0)		−0.0989***	−0.101***	−0.101***
		(0.0151)	(0.0151)	(0.0151)
Firm scale		−0.0971***	−0.0969***	−0.0967***
		(0.00864)	(0.00864)	(0.00864)
Fixed effects for subindustries	No	Yes	Yes	Yes
Fixed effects for cities	No	Yes	Yes	Yes
Constant	2.891***	1.074***	0.938***	0.803***
	(0.0154)	(0.109)	(0.118)	(0.125)
Observations	28,470	28,470	28,470	28,470
*R*-squared	0.010	0.063	0.063	0.063

Significance level: ***p < 0.01, **p < 0.05, *p < 0.1.

Model 3 also reports the associations between sociodemographic variables and PPFs. First, gender and PPFs are positively correlated (*p* < 0.01). Consistent with Kuhn (1987) and Pfeifer and Stephan (2018), this result suggests that, all else being equal, female workers are more likely than their male counterparts to perceive their pay as fair. Second, worker age is significantly positively associated with PPFs (*p* < 0.05), indicating that older workers are more likely to view their pay as fair compared to younger workers. Third, employment tenure is negatively linked to PPFs (*p* < 0.01), with workers who have been employed longer perceiving their pay as less fair. Fourth, education shows a significant negative association with PPFs (*p* < 0.01), with higher-educated workers more likely to feel their pay is unfair. Meanwhile, *hukou* status is not significantly associated with workers’ PPFs.

Additionally, Model 3 reveals the correlations between job-level variables and PPFs. As expected, relative hourly wages are positively related to workers’ PPFs (*p* < 0.01), indicating that workers with higher wages are more likely to perceive their pay as fair compared to those with lower wages. Additionally, workers in privately owned or foreign-owned firms (including those in Hong Kong, Taiwan, or Macao) report significantly lower PPFs compared to those in state-owned firms, with differences significant at the 1% level. Firm size is significantly negatively associated with PPFs (*p* < 0.01), as workers in larger firms tend to perceive their pay as less fair compared to workers in smaller firms.

Model 4 introduces the interaction term between robotization and gender. The coefficient for this interaction is −0.0377 (*p* < 0.05), indicating that the positive association between robotization and PPFs is less pronounced for female workers. This finding suggests that although robotization are generally associated with a greater likelihood of workers perceiving their pay as fair, there is a significant gender disparity in this association. Specifically, male workers show a stronger positive association between robotization and PPFs compared to female workers. These results provide support for Hypothesis 2.

### 4.2 Instrumental variable (2SLS) estimation and robustness checks

To strengthen the causal inference in our analysis, we employed a two-stage least squares (2SLS) regression using an instrumental variable (IV) approach (Table 3). Given the potential endogeneity in the relationship between robotization and PPF, and to address the complexity introduced by gender interactions, we performed separate 2SLS regressions for male and female subsamples. This allowed us to compare the magnitude of the robotization effect across genders and to address possible endogeneity in models with interaction terms.

**Table 3:** The impact of robotization on PPFs by gender (IV-2SLS results).

Variables	Man		Woman		Total Sample	
	(1)	(2)	(3)	(4)	(5)	(6)
	First stage	Second stage	First stage	Second stage	First stage	Second stage
Robotization-IV	0.0575***		0.060***		0.0700***	
	(0.014)		(0.017)		(0.0120)	
Robotization		0.927**		0.619*		0.801***
		(0.376)		(0.357)		(0.246)
Constant	0.592***	0.766**	0.673***	0.636**	0.760***	0.463*
	(0.141)	(0.336)	(0.161)	(0.349)	(0.109)	(0.266)
Observations	13,000		10,266		25,323	
*F*	16.36		12.05		33.84	

1. In the model, variables at both the individual and firm levels are controlled, along with fixed effects for industry and city groups. Because of space constraints, the results for the control variables are not presented in the model. 2. Significance level: ***p < 0.01, **p < 0.05, *p < 0.1.

To construct the IV for robotization, we drew on firm and local context: specifically, for each worker, the instrument was defined as the average reported level of robotization among all other firms in the same industry, of similar size, and located in the same city (excluding the worker’s own firm). The rationale is that a firm’s likelihood of adopting robots is influenced by broader industry trends, size-related technological capacity, and local diffusion of automation, but the adoption decisions of other firms should not directly affect individual PPF, aside from their influence on robot adoption in the respondent’s own workplace. All other covariates from the baseline model were retained.

The 2SLS results reinforce our main findings. For the overall sample, robotization has a significant positive effect on workers’ PPFs. When disaggregated by gender, the coefficient for robotization on PPFs is 0.927 for male workers and 0.619 for female workers, indicating a substantial difference in the strength of the effect. This result is consistent with our baseline OLS estimates and provides further support for the conclusion that robotization exerts a stronger positive influence on perceived pay fairness among male workers compared to female workers (Table 3).

Furthermore, we conducted a series of robustness checks. First, we used ordered logit and ordered probit models as alternative estimation strategies to assess the robustness of our main findings. As shown in Models (1) and (2) of Table 4, the results from both models are consistent with those of the baseline analysis, with the estimated coefficients maintaining their direction, magnitude, and statistical significance.

**Table 4:** Robustness checks.

Variables	Model (1): Ordered logit	Model (2): Ordered probit	Model (3): Robotization (No/Yes)	Model (4): FIML
Robotization	0.454***	0.241***	0.340***	0.324***
	(0.0473)	(0.0265)	(0.0427)	(0.0686)
Female (male = 0)	0.284***	0.153***	0.0499***	0.272***
	(0.0642)	(0.0362)	(0.0125)	(0.0838)
Robotization × Female	−0.107***	−0.0548***	−0.0919***	−0.121***
	(0.0309)	(0.0175)	(0.0287)	(0.0428)
Constant			1.057***	0.525***
			(0.122)	(0.177)
Observations	28,470	28,470	28,470	28,470

1. In the model, variables at both the individual and firm levels are controlled, along with fixed effects for industry and city groups. Because of space constraints, the results for the control variables are not presented in the model. 2. Significance level: *** *p* < 0.01.

Next, we redefined the robotization variable as a binary indicator—distinguishing between workshops with and without robot usage—and re-estimated the model. As shown in Model (3) of Table 4, the results remained robust and consistent with our primary findings.

Finally, to address potential concerns regarding sample selection bias, we applied the full information maximum likelihood (FIML) method. The FIML model yielded a non-significant *ρ*₁₂, suggesting that our sample was not subject to substantial selection bias, and the estimates remained consistent with our main analysis.

Overall, these robustness checks further confirm the stability of our results—specifically, that the interaction term between robotization and gender is consistently negative and significant at the 1% level, underscoring the persistent gender disparity in the impact of robotization on perceived pay fairness.

### 4.3 How robotization’s impact on PPFs differs by gender

In this section, we systematically test Hypotheses 3–6 regarding the gender-specific mediating effects of wages and skill levels in the relationship between robotization and PPF. Our analytical strategy is as follows: we begin by describing the distributions of wages and skill levels among male and female workers. Next, we estimate separate regression models for men and women to examine the effect of robotization on these mediators. Finally, we employ bootstrap mediation analysis to decompose the total effect of robotization on PPFs into direct and indirect pathways. All models include the same set of control variables (including individual and firm characteristics, industry, and city fixed effects) as in the baseline regression.

#### 4.3.1 Mediating effect of wages

In this section, we test Hypothesis 3 and 4 by investigating the mediating roles of wages in the relationship between robotization and PPFs for male and female workers.

**Figure 1:**
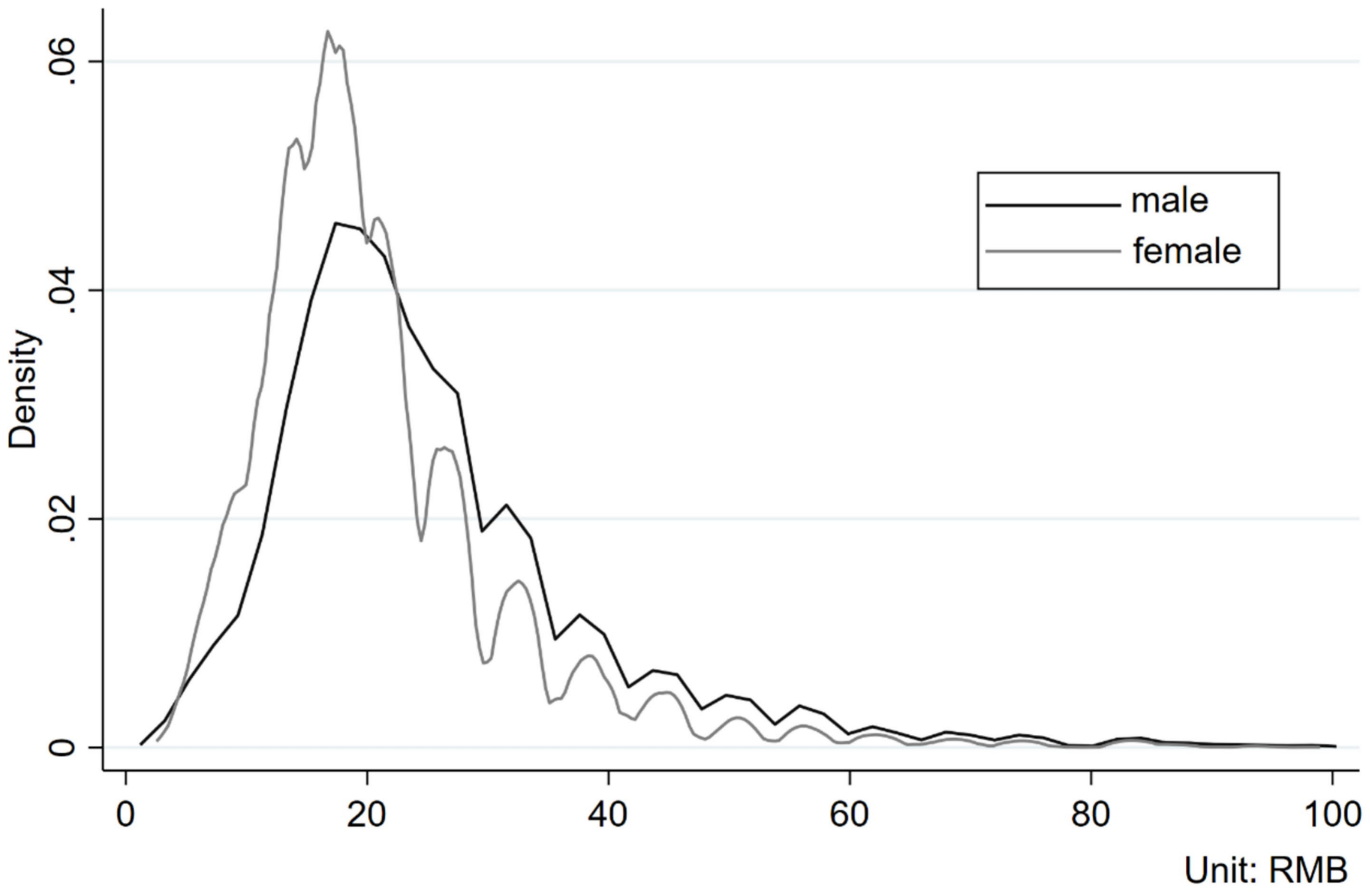
Density plot comparing the hourly wages of male and female.

First, Figure 1 displays density plots of the hourly wage distribution for men and women. The data show that, in the segment where hourly wages are less than RMB20 (approximately USD2.97), the density of the female distribution exceeds that of the male distribution. Conversely, beyond the RMB20 threshold, the density of the male distribution surpasses that of the female distribution. This indicates that women are concentrated in lower wage brackets, while men are more represented in higher wage brackets. Additionally, survey data reveal that the average hourly wage for men is RMB27.16 (approximately USD4.04), while for women is RMB22.12 (approximately USD3.29), representing a difference of 18.57%. This finding is consistent with those of studies in China (e.g., Zhang et al., 2008; Li et al., 2011), indicating that men’s wages are higher than those of women.

Table 5 presents the regression results on the impact of robotization on the hourly wages for male and female workers. The findings indicate that robotization significantly increases wages for both genders (both *p* < 0.01). However, the coefficient for men (0.0424) is larger than that for women (0.0255), indicating the wage increase associated with robotization is more pronounced for men. Additionally, the Fisher’s Permutation Test reveals a statistically significant difference in the impact of robotization on the wages of men and women (*p* < 0.05).

**Table 5:** Effects of robotization on men’s and women’s hourly wages.

Variable	Model (1): Lnhwage (women)	Model (2): Lnhwage (men)	Model (3): Lnhwage (total sample)
Robotization	0.0255***	0.0424***	0.0341***
	(0.00525)	(0.00497)	(0.00361)
Constant	1.694***	1.714***	1.892***
	(0.0419)	(0.0399)	(0.0303)
Observations	12,766	15,704	28,470
*R*-squared	0.251	0.206	0.248
Intergroup differences	−0.017**		

1. In the model, variables at both the individual and firm levels are controlled, along with fixed effects for industry and city groups. Because of space constraints, the results for the control variables are not presented in the model. 2. Significance level: *** *p* < 0.01, ** *p* < 0.05. 3. The *p*-values for intergroup difference testing are derived from Fisher’s Permutation Test with 1,000 sampling iterations.

The next stage of our analysis examines the mediating role of hourly wages in the relationship between robotization and PPFs for both men and women, as shown in Tables 6, 7. Bootstrap analysis confirms that hourly wages significantly mediate the positive effect of robotization on PPFs for both genders. Notably, the indirect effect is larger for men (0.0166) than for women (0.00991), indicating that wage increases play a stronger mediating role for men than for women in this context.

**Table 6:** Mediating effects of hourly wage on the impact of robotization on the PPFs of men (Bootstrap test).

Type of effect	Coefficient	Boot S.E.	95% conf. interval
Total effect	0.166***	0.011	0.145–0.188
Indirect effect	0.0166***	0.00225	0.0122–0.0210
Direct effect	0.15***	0.011	0.128–0.171

1. In the model, variables at both the individual and firm levels are controlled, along with fixed effects for industry and city groups. Because of space constraints, the results for the control variables are not presented in the model; 2. Significance level: *** *p* < 0.01; 3. The mediating effect was tested using bias-corrected bootstrapping with 1,000 resamples.

**Table 7:** Mediating effects of hourly wage on the impact of robotization on the PPFs of women (Bootstrap test).

Type of effect	Coefficient	Boot S.E.	95% conf. Interval
Total effect	0.117***	0.0112	0.095–0.139
Indirect effect	0.00991***	0.00219	0.00563–0.0142
Direct effect	0.107***	0.011	0.0855–0.129

1. In the model, variables at both the individual and firm levels are controlled, along with fixed effects for industry and city groups. Because of space constraints, the results for the control variables are not presented in the model; 2. Significance level: *** *p* < 0.01; 3. The mediating effect was tested using bias-corrected bootstrapping with 1,000 resamples.

These results support Hypothesis 3, which predicts that wages mediate the positive effect of robotization on PPFs. However, Hypothesis 4—which posited that women’s higher satisfaction with smaller wage growth would offset the gender difference—is not supported; instead, the wage-based mediation effect is weaker for women.

#### 4.3.2 Mediating effect of skill levels

In this section, we test Hypothesis 5 and 6 by investigating the mediating roles of skill levels in the relationship between robotization and PPFs for male and female workers.

**Figure 2:**
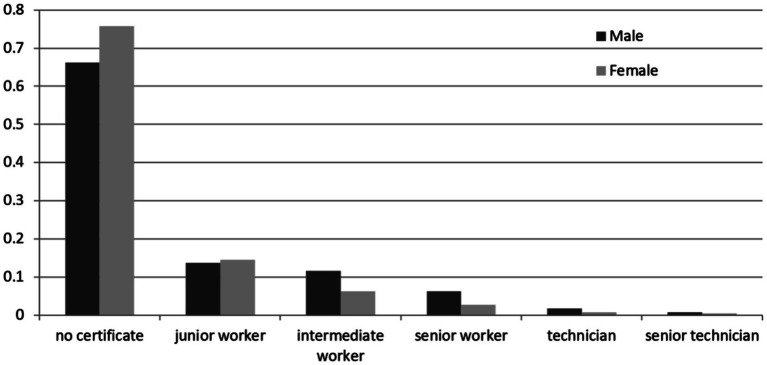
Distribution of occupational skill levels by gender.

First, Figure 2 shows the distribution of occupational skill levels by gender. On average, male workers have higher skill levels than their female counterparts, with men scoring 1.65, surpassing women by 0.29. Furthermore, the proportion of female workers without skill certificates exceeds that of male workers by about 10 percentage points. While women slightly outnumber men at the junior worker certification level, men dominate at all other skill levels, including intermediate worker, senior worker, technician, and senior technician.

Second, Table 8 presents the regression results on the impact of robotization on the skills of male and female workers. The findings indicate that robotization significantly enhances skills for both genders (both *p* < 0.01). Moreover, Fisher’s Permutation Test suggests no significant difference between the impact of robotization on skills for men and women.

**Table 8:** Effects of robotization on men’s and women’s skills.

Variable	Model (1): Skills (women)	Model (2): Skills (men)	Model (3): Skills (total sample)
Robotization	0.0548***	0.0491***	0.0572***
	(0.0094)	(0.0112)	(0.0075)
Control variables	Yes	Yes	Yes
Fixed effects for subindustries	Yes	Yes	Yes
Fixed effects for cities	Yes	Yes	Yes
Constant	0.213***	0.273***	0.571***
	(0.0766)	(0.0928)	(0.0649)
Observations	12,766	15,704	28,470
*R*-squared	0.104	0.114	0.126
Intergroup difference	0.011		

1. In the model, variables at both the individual and firm levels are controlled, along with fixed effects for subindustry and city groups. Because of space constraints, the results for the control variables are not presented in the model. 2. Significance level: *** *p* < 0.01. The *p*-values for intergroup difference testing are derived from Fisher’s Permutation Test with 1,000 sampling iterations.

Furthermore, we analyze the mediating role of skills in the robotization-PPF relationship among men and women, as shown in Tables 9, 10. For the male group, skills act as a mediating factor that weakens the positive effect of robotization on PPFs (*p* < 0.01) (see Table 9). In contrast, for the female group, the mediating effect of skills is not statistically significant in the robotization-PPF relationship (see Table 10).

**Table 9:** Mediating effects of skills on the impact of robotization on PPFs among men (Bootstrap test).

Type of effect	Coefficient	Boot S.E.	95% conf. Interval
Total effect	0.166***	0.0107	0.145–0.187
Mediating effect	−0.000785***	0.000417	−0.00160−−0.0000318
Direct effect	0.167***	0.0107	0.146–0.188

1. In the model, variables at both the individual and firm levels are controlled, along with fixed effects for subindustry and city groups. Because of space constraints, the results for the control variables are not presented in the model; 2. Significance level: *** *p* < 0.01. The mediating effect was tested using bias-corrected bootstrapping with 1,000 resamples.

**Table 10:** Mediating effects of skills on the impact of robotization on PPFs among women (Bootstrap test).

Type of effect	Coefficient	Boot S.E.	95% conf. Interval
Total effect	0.117***	0.0127	0.0921–0.142
Mediating effect	0.00081	0.000754	−0.000670–0.00229
Direct effect	0.116***	0.0128	0.0910–0.141

1. In the model, variables at both the individual and firm levels are controlled, along with fixed effects for subindustry and city groups. Because of space constraints, the results for the control variables are not presented in the model; 2. Significance level: *** *p* < 0.01; 3. The mediating effect was tested using bias-corrected bootstrapping with 1,000 resamples.

These findings support Hypothesis 5: for men, increased skill levels due to robotization are associated with higher wage expectations, which weakens the positive effect of robotization on PPFs. Hypothesis 6 is also supported: skill improvements do not significantly mediate the robotization–PPF link among women.

## 5 Discussion

This study investigates the gender-specific impacts of robotization on PPFs in the Chinese manufacturing industry. Our findings show that robotization is a significant factor in shaping workers’ PPFs. Workers in robotized workplaces generally perceive their pay as fairer than those in non-robotized environments, even when wages remain similar. Additionally, the study demonstrates that gender moderates this relationship: while robotization positively affects PPFs for both genders, the impact is weaker for female workers compared to their male counterparts. To the best of our knowledge, this is the first study to explore the impact of robotization on workers’ PPFs, in particular from a gender perspective.

One of the key explanations for this gender disparity lies in the differing impacts of robotization on men and women’s wages. Although robotization is associated with wage increases for both genders, men experience greater wage growth than women, a finding consistent with prior research (Aksoy et al., 2020; Pavlenkova et al., 2023). The smaller wage increase experienced by women correspond to a more limited improvement in their PPFs, suggesting that women are not necessarily more satisfied with smaller wage gains from robotization. Instead, the limited wage growth they experience is linked to a weaker positive impact on their PPFs. Consequently, the difference in gender wage growth contributes to the gender disparity in PPFs.

Additionally, the study reveals differing mediating roles of skill levels in the robotization-PPF relationship for men and women. For male workers, robotization raises skill levels, but this also weakens the positive impact of robotization on PPFs. This likely occurs because men, with greater confidence in their skills, tend to increase their wage expectations as their skills improve, thereby reducing the positive effect of robotization on their PPFs. In contrast, although robotization also improves skill levels for female workers, it does not significantly affect their PPFs. One of the possible explanations is that gender norms and workplace structures often lead women to undervalue their own skills and set lower reference points for fair pay, even when their objective skill levels rise. The phenomenon of “internalized undervaluation” means that skill growth does not prompt women to adjust their wage expectations upward to the same extent as men. As a result, the psychological mechanism whereby increased skills would lead to perceived under-compensation—and thus reduced fairness—does not activate as strongly for women. Furthermore, the lack of significant mediation for women may also reflect a “ceiling effect” rooted in gendered organizational practices. In environments where female workers already perceive a structural cap on their advancement or pay, further skill acquisition may not meaningfully alter their reference group or fairness perceptions, because they do not expect their skills to be fairly rewarded in the first place. This interaction between internalized gender norms and structural barriers helps explain why skill enhancement fails to significantly mediate the effect of robotization on perceived pay fairness among female workers.

In summary, compared with females, robotization enhances male workers’ Perceived Pay Fairness (PPFs) to a greater extent. This is mainly attributed to the wage mechanism. Although the skill mechanism slightly weakens the positive effect of robotization on male workers’ PPFs, the wage mechanism exerts a more substantial influence among the two mechanisms. Consequently, robotization ultimately demonstrates a stronger promoting effect on male workers’ PPFs. Although the skill mechanism does not decrease female workers’ PPFs, given that the wage mechanism is more crucial and female workers have much lower wage growth rates compared to male workers, the effect of robotization in enhancing female workers’ PPFs is significantly lower than that for male workers.

Based on these findings, we offer two key recommendations. First, governments and human resource professionals should address the wage disparities caused by robotization, particularly their impact on women’s PPFs. Our study shows that when women’s wage growth lags behind men’s, their PPFs are also lower, highlighting women’s sensitivity to wage changes resulting from robotization. To close the gender gap in PPFs, policymakers and employers should ensure that robotization contributes equally to wage growth for both genders. Second, we recommend providing training for female workers to dismantle gender stereotypes in robotics and boost their confidence in their skills. Research indicates that PPFs play a critical role in maintaining gender wage disparities (Pfeifer and Stephan, 2018); when women undervalue their skills in robotized environments, they may be less likely to negotiate for higher wages, perpetuating gender wage gaps. Therefore, targeted training to help women accurately assess their skills in robotized work settings could promote a more equitable wage structure.

This study has several limitations. First, the measurement of key variables may affect the robustness of the findings. PPF was assessed using a single-item Likert scale. Although this approach reduces participant burden and simplifies data collection, it may lack the reliability and validity offered by multi-item scales. Future research is encouraged to employ more comprehensive and validated multi-item measures to further test and refine these results. Similarly, skill levels were measured based on self-reported certificate data rather than objective skills assessments, which may introduce measurement error. Future studies should consider using more objective and detailed indicators of skill to better capture this relationship. Second, the online, snowball-style sampling method may introduce measurement bias and sampling bias. Although robustness checks using the Full Information Maximum Likelihood (FIML) method indicated no severe sampling bias, future studies should adopt more rigorous sampling procedures to reduce potential bias. Third, it examines the overall impact of robotization on workers’ PPFs but does not account for how individual workers’ direct interaction with robots might influence their PPFs. Future research could explore how factors such as the frequency and intensity of robot use shape PPFs. In addition, combining subjective and objective measures of robotization—for example, using both self-reported exposure and objective indicators such as robot density—could provide a more comprehensive assessment of how robotization affects workers’ PPFs. Fourth, the data were collected exclusively from Guangdong province, which may limit the generalizability of the findings to other regions in China. Future studies could expand the geographic scope or conduct cross-region comparisons to gain a broader understanding of the impact of robotization on PPFs.

## 6 Conclusion

Using survey data from 28,470 workers in Guangdong province, this study provides new insights into the gendered effects of robotization on workers’ PPFs in the Chinese manufacturing industry. The findings indicate that while robotization is generally associated with higher PPFs among workers, this association is stronger for men, largely reflecting gender wage disparities. Men tend to experience greater wage growth in connection with robotization, which is linked to more pronounced improvements in their PPFs. In contrast, women’s more limited wage growth corresponds to a weaker association with PPFs. Additionally, although robotization is associated with improvements in skill levels for both genders, increased skill levels appear to reduce the positive association between robotization and PPFs among men, while no significant association is observed among women. Differences in skill confidence and wage expectations between men and women may help explain these variations in the mediating roles of skill levels.

## Data Availability

The raw data supporting the conclusions of this article will be made available by the authors, without undue reservation.

## Footnotes

^1^*Hukou* is the household registration system in mainland China, which distinguishes individuals living in agricultural (rural *hukou*) and nonagricultural areas (urban *hukou*). Research suggests that migrant workers in urban areas with rural *hukou* typically experience labor market discrimination (Song, 2014). Therefore, we include a *hukou* variable in the regression analysis.

^2^Small firms have fewer than 100 employees, medium-sized firms have 100–999 employees, and large firms have 1,000 or more employees.

^3^Firms were classified into three categories based on the number of employees: small firms with 1–99 employees, medium-sized firms with 100–999 employees and large firms with 1,000 or more employees.
